# The psychometric properties of the Persian version of the moral injury symptoms scale-health care professionals version

**DOI:** 10.3389/fpsyg.2022.978572

**Published:** 2022-08-05

**Authors:** Alireza Malakoutikhah, Mohammad Ali Zakeri, Harold G. Koenig, Mahlagha Dehghan

**Affiliations:** ^1^Student Research Committee, School of Nursing and Midwifery, Kerman University of Medical Sciences, Kerman, Iran; ^2^Non-Communicable Diseases Research Center, Rafsanjan University of Medical Sciences, Rafsanjan, Iran; ^3^Social Determinants of Health Research Centre, Rafsanjan University of Medical Sciences, Rafsanjan, Iran; ^4^Department of Psychiatry and Behavioral Sciences, and Department of Medicine, Duke University Medical Center, Durham, NC, United States; ^5^Department of Critical Care Nursing, Razi Faculty of Nursing and Midwifery, Kerman University of Medical Sciences, Kerman, Iran

**Keywords:** validation, moral injury, health care, reliability, psychometric

## Abstract

**Background:**

Health care professionals face a number of problems during crises, such as the COVID-19. Studies addressed the prevalence of moral injury among healthcare professionals during the COVID-19 outbreak. Lack of a valid standard of moral injury among health care professionals is one of the factors that has made it difficult to identify and treat this complication. This study aimed to evaluate the psychometric properties of the Moral Injury Symptoms Scale-Health Care Professionals (MISS-HP) among health care professionals in Iran.

**Methods:**

This study was conducted to evaluate the validity and reliability of the MISS-HP. The sample included 455 healthcare professionals working in four teaching hospitals in Kerman, who were in direct contact with patients. In this study, face validity, content validity, construct validity (structural and convergent), and internal reliability of the MISS-HP were evaluated. Demographic information questionnaire, the Moral Injury Symptoms Scale-HealthCare Professionals (MISS-HP), General Health Questionnaire (GHQ), and Impact of Event Scale (IES) were administered to study participants.

**Results:**

The MISS-HP was evaluated using translation-back translation technique. The content validity index of the items (I-CVI) and the scale (S-CVI) were 0.9 and 0.99, respectively. Exploratory factor analysis showed a three-factor structure in the MISS-HP that explained 57.49% of the variance. Confirmatory factor analysis indices were acceptable. The cut-off point of the questionnaire was 36.5. There was a positive and moderate correlation between the Persian version of MISS-HP, GHQ (*r* = 0.34), and IES-R (*r* = 0.40). The Cronbach’s alpha coefficient of the Persian version of MISS-HP was 0.70.

**Conclusion:**

This study found that the MISS-HP is a concise, comprehensive, valid and reliable scale for assessing moral injury among health care professionals in clinical or research settings. This scale will be helpful for managers and researchers to identify and plan health policies and improve the psychological state of health care professionals.

## Introduction

It seems easy to adhere to moral values, but it has its own difficulties and complexities. Oxford dictionary defines morality as “principles concerning the distinction between right and wrong or good and bad behavior” (Stevenson, 2010). Moral values are cultural values transmitted primarily through family members, peer groups, particularly friends, or religious beliefs formed by religious organizations (Feldman, 2021).

When people encounter moral conflicts in everyday situations, they tend to resolve them according to their own personal values, so they avoid transgressions (Haidt, 2012). It is often difficult, if not impossible, to know the morally correct course of action. Part of the reason is that it is difficult to determine a correct moral theory or moral principle (Campbell et al., 2018). Given the wide range of possible actions we can take and their consequences in the medical situations, it is not unexpected that we often do not know which behaviors are morally correct. When we lack such knowledge, moral distress arises—even in cases where we know exactly what morality demands of us. In such situations, distress can occur in the form of guilt or self-criticism (Campbell et al., 2018). Moral distress has become a well-known topic in the nursing literature and is receiving more and more attention in other areas of health care. Moral distress arises when one knows the right thing to do, but institutional constraints make it nearly impossible to pursue the right course of action (Oh and Gastmans, 2015).

In addition, the experience of moral dilemma is not limited to any professions or situations. Military personnel also experience or witness events such as facing intense human suffering and witnessing the results of their actions. For example, military medics are often asked to prioritize caring for some casualties over others, which can be experienced as “choosing who survives and who dies” (Bryan et al., 2016). The term “moral injury” was coined by Shay et al. based on veteran patients who perceived injustice as a result of leadership malpractice. According to Shay “Moral injury is present when (1) there has been a betrayal of what is morally right; (2) by someone who holds legitimate authority; and (3) in a high-stakes situation” (Shay and Munroe, 1999). Litz, by focusing on the individual and feelings of self-betrayal, defined moral injury as perpetuating, failing to prevent, bearing witness to, or learning about acts that transgress deeply held moral beliefs and expectations’ (Litz et al., 2009).

Furthermore, other researchers tried to use alternative terms for “injury” that generally refers to physiological damage or the word “moral” including “moral affront” (Neilson, 2015), “moral distress” (Corley et al., 2001; Musto et al., 2015), “moral conflict” (Hale, 2013), “moral pain” (Verkamp, 2006), “moral trauma,” “moral wounds,” “moral disruption,” and “emotional injury,” “personal values injury,” “life values injury,” and “spiritual injury” (Drescher et al., 2011). As Phelp’s et al. (2015) noted, there is “no agreed definition of moral injury.” In addition, Kopacz et al. (2014a,b) believes that “moral injury remains a relatively abstract concept that is still in its empirical infancy, with as yet undetermined applicability in clinical, public health, or research settings.”

The World Health Organization (WHO) has also identified work-related stress as one of the various health risks and a global hazard (Leka et al., 2003). Meanwhile, healthcare professionals will be mostly vulnerable, especially those who are caring for injured patients (Zakeri et al., 2021c; Bazmandegan et al., 2022). Medical staff work in stressful environments and often experience long or stressful working hours, which can impair the mental and emotional functioning of medical staff (Hossini Rafsanjanipoor et al., 2021; Zakeri et al., 2021a). On the other hand, crises exacerbate emotional problems (Zakeri et al., 2021d). A study by Huang et al. during the COVID-19 epidemic in 2020 showed that the closer the disease was to health care providers, the greater was their anxiety and anger (Huang et al., 2020). Zakeri et al. also showed that 48.2% of healthcare professionals reported psychological disorders in the outbreak of COVID-19 (Zakeri et al., 2021b). These findings indicate the emotionally devastating effects of crises on health care professionals, leading to individual challenges (Huang et al., 2020).

Decisions made under such critical circumstances can violate basic medical care guidelines on the front lines and can expose some health care professionals to moral injury/distress (Protopopescu et al., 2020). People with moral conflicts may suffer from moral injury or distress, which may have a significant impact on social, occupational, and family performance (Koenig et al., 2019). Griffin et al. (2019) conducted a review study and demonstrated that moral injury/distress could lead to a breakdown of social bonds, negative changes in mental development, and other functional disorders.

Lack of a valid measure of moral injury/distress among health care professionals has slowed down the identification and treatment of this complication. By identifying moral injury and its symptoms, health systems and organizations can hold training and intervention programs to address moral injury that affects the safety and security of health care professionals (Stovall et al., 2020). Despite significant advances in identifying and treating moral injury among military personnel (Koenig et al., 2019), there were few studies on healthcare personnel (Mantri et al., 2020). Long (Koenig et al., 2018a) and short forms (Koenig et al., 2018b) of Moral Injury Symptom Scale-Military were designed to identify and measure the signs of moral injury in veterans and active-duty military personnel. Moral Injury Symptom Scale-Healthcare Professionals Version is the only version developed and evaluated by Mantri et al. for a valid measurement of moral injury in health care professionals. This scale is used to identify symptoms affecting social, occupational, and functional problems among health care professionals, who are at high risk of moral injury (Mantri et al., 2020). Considering the importance of recognizing and measuring moral injury in health care professionals and a lack of Persian measuring tool in this field, the present study aimed to investigate the psychometric properties of the Persian Version of the Moral Injury Symptoms Scale-Health Care Professionals Version.

## Materials and methods

### Study design and setting

This cross-sectional study consisted of two phases: forward and backward translation of the MISS-HP into Persian and then determination of the psychometric properties of the MISS-HP-Persian version. The research setting was four teaching hospitals affiliated to Kerman University of Medical Sciences in southeast Iran.

### Participants, sample size, and sampling

The study population was a convenience sample of medical staff from four hospitals in Kerman. The inclusion criteria were healthcare professionals who had at least 1 year of work experience. Exclusion criteria were: (1) Failure to complete the questionnaire for any reason (incomplete completion of more than 10% of the number of questions in each questionnaire), (2) History of mental illness leading to hospitalization or long-term use of psychiatric drugs (self-reported).

The number of participants for each phase of the study was as follows: (1) qualitative face validity: 10 healthcare professionals, (2) quantitative face validity: 10 healthcare professionals, (3) qualitative content validity: 10 experts, (4) quantitative content validity: 10 experts, (5) pilot study (for checking internal consistency before conducting exploratory factor analysis): 50 healthcare professionals, (6) exploratory factor analysis (EFA): 255 healthcare professionals, (7) confirmatory factor analysis (CFA): 200 healthcare staff, and (8) convergent validity: 455 healthcare professionals.

In addition, questionnaires from 33 participants were excluded for not meeting the inclusion criteria, having confounding information, and missing values. The study took place between September 23, 2021, and February 1, 2022.

### Measures

#### Demographic characteristics form

Demographic characteristics form included information such as age, gender, marital status, level of education, academic major, income, work experience (month), name of hospital and ward, and having a mental disorder (yes/no).

#### The moral injury symptom scale-healthcare professionals version

MISS-HP designed and used by Mantri et al. in the Covid-19 outbreak in 2020, assesses the symptoms of moral injury in healthcare professionals. Its internal reliability in the study of Mantri et al. was 0.75. Principal Component Factor Analysis also identified three factors for this scale, which was confirmed by confirmatory factor analysis. Divergent validity had a moderate correlation with symptoms of religiosity, depression, and low anxiety (*r* = 0.25–0.37), while the convergence validity was strongly correlated with burnout (*r* = 0.57). All 10 MISS-HP items have visual analog scale response options ranging from 1 (strongly disagree) to 10 (strongly agree). In order to reduce the response bias, four positive items and six negative items are presented. After coding items (5, 6, 7, and 10) positively, items scores are added up to an overall score of 10–100, with higher scores indicating further moral injury. On the end of the scale, there is a question (item number 11), examining the emotions expressed in the previous 10 items in general, and how much they have disturbed the ability to function in relationships, the workplace or other areas of life, which is answered with not at all, mild, moderate, very much, extremely (Mantri et al., 2020).

#### The general health questionnaire-12

The General Health Questionnaire (GHQ) was developed by Goldberg in 1972 to assess mental disorders in a variety of settings (Goldberg, 1972). This self-report questionnaire consists of 60 items, the short forms of which are also available in 12, 20, 28, and 30 items. GHQ examines a person’s mental state in a month before (Fryers et al., 2004). This study used the 12-item short form. The GHQ-12 serves as a simple and rapid screening tool to identify individuals with minor psychological disorders, who are at risk for psychiatric disorders (Goldberg and Williams, 1988). The GHQ-12 internal consistency reliabilities ranged from 0.70 to 0.91. In addition, the test-retest reliabilities were reported to be 0.84 after 7–14 days and 0.79 after 20 days (Gnambs and Staufenbiel, 2018). According to Namjoo et al., the content validity index and content validity ratio of the GHQ-12 in an Iranian population were 0.92 and 0.96, respectively. Najarkolaei et al. also reported the Cronbach’s alpha coefficients to be 0.82 (Namjoo et al., 2017) and 0.85 (Najarkolaei et al., 2014). The questionnaire is answered based on a four-point Likert scale. In this method, the minimum and maximum scores for the 12-item short form will be 0 and 36, with a higher score indicating a higher mental disorder (Donath, 2001). The present study used the GHQ-12 to evaluate the convergent validity.

#### Impact of event scale-revised

The IES was designed by Horowitz et al. in 1979 to assess the psychological impact of an event. This scale was designed to determine two patterns of psychological response to trauma, including signs of avoidance, withdrawal and curiosity (Horowitz et al., 1979). Weiss et al. revised the IES in 1997 (Weiss and Marmar, 1997). The IES is a valid measure for assessing post-traumatic stress disorder. Creamer et al. confirmed its validity and reliability and reported a Cronbach’s alpha of 0.96 (Creamer et al., 2003). Abdi et al. (2010) reported the internal consistency to be between 0.79 and 0.92, and they found a good reliability for it. The present study used the IES-R to evaluate the convergent validity.

### Procedure, data collection, and data analysis

#### Forward and backward translation of moral injury symptoms scale-health care professionals

First, the Moral Injury Symptoms Scale-Health Care Professionals Version was translated into Persian by two Farsi-language translators, one of whom was familiar with the medical concepts. Then, another Farsi-language translator combined the translations. In the next phase, two English-language translators did the backward translation to English again. Given that the Persian version should be semantically, idiomatically, experiential, and conceptually equivalent to the original version, the research team and translators made the final editing’s in the Persian version if necessary. The backward translation was checked by Dr. Harold G. Koenig (the original scale developer) and some modifications were made (items #3, #6, and #9) on the Persian version according to his comments.

#### Face validity

Both qualitative and quantitative methods were used to determine the face validity.

For qualitative face validity, the researchers conducted face to face interviews with 10 samples (seven nursing staff and three physicians), and difficulty levels (difficulty in comprehending words and sentences), relativity (appropriateness and relation of sentences with the inventory dimensions), and ambiguity (probability of misinterpretations of expressions or the inaccuracy of word meanings) were examined.

For quantitative face validity, the Item Impact Method was used to determine the importance of each phrase. In this method, the proportion of participants who rated the item as significant (frequency in percentage) was multiplied by the mean score of the item importance.

Significance (Mean) × Frequency (%) = Item Impact Score

In the Item Impact Method, if the impact score is equal to or greater than 1.5, the phrase will be appropriate for subsequent analysis. At this stage, the scale was provided to 10 health care workers (Heravi-Karimooi et al., 2010; Shahhosseini et al., 2011).

#### Content validity

To determine the content validity of the scale in this study, both qualitative and quantitative methods were used. In the qualitative evaluation of the content validity, the scale was provided to experts including medical and nursing faculty members, psychologists, and methodologists. They were requested to write down their opinions on content coverage, grammar compliance, use of the right phrases, and the right place of the items. In the quantitative evaluation of the content validity, Content Validity Index (CVI) was used. Experts were asked to determine CVI to examine each item on a four-point scale (1 = not relevant, 2 = requires major review, 3 = relevant but needs minor review, 4 = completely relevant). The Item-Content Validity Index score (I-CVI) was calculated by dividing the number of experts agreeing with numbers 3 and 4 by the total number of experts. If I-CVI was 0.8 or greater, its validity was accepted. In addition, to calculate the Scale-Content Validity Index (S-CVI), the mean score of I-CVI of all items was calculated. If the S-CVI of the scale was 0.9 or greater, it was acceptable (Heravi-Karimooi et al., 2010).

#### Construct validity

##### Structural validity

For structural validity, both exploratory and confirmatory factor analyses were conducted. In exploratory factor analysis, all Principal Component analysis (PCA), Principal Axis Factoring, and Maximum Likelihood were used to extract the factors (structures). The Varimax and Promax Rotation methods were used to rotate the items. The following criteria were used to determine the number of factors: (a) eigenvalues >1, (b) scree plots, (c) items with loadings of 0.4 or greater on any one factor (Costello and Osborne, 2005; Chehrei et al., 2016). Finally, the best method was PCA extraction with Promax Rotation. Confirmatory factor analysis was used to assess the structure of the factors derived from exploratory factor analysis. The model’s adequacy was determined using the chi squared test. CMIN, Goodness-of-Fit Index (GFI), Adjusted Goodness-of-Fit Index (AGFI), Comparative Fit Index (CFI), Incremental Fit Index (IFI), Normed Fit Index (NFI), and Root Mean Squared Error of Approximation (RMSEA) are the main indices used to determine the fit of the model. Acceptable fit of the model was indicated by χ^2^/df < 3.0, and RMSEA < 0.08. The GFI, AGFI, CFI, IFI, and NFI indices all had values of ≥0.9 (Jay Lynn et al., 2006).

SPSS25 was used to fit the exploratory factor analysis model to the data and AMOS24 to fit the confirmatory factor analysis model.

##### Determination of cut-off point

According to Mantri et al. (2020) study, participants were asked right after they finished the MISS-HP: “Do the feelings you listed above cause you significant distress or make it hard for you to function in relationships, at work, or other areas of life important to you? In other words, if you listed any problems above, how hard has it been for you to do your work, take care of things at home, or get along with other people because of these problems?” Response options were in the forms of “not at all,” “mild,” “moderate,” “very much,” and “extremely.” The symptoms that caused functional disability were put into two groups: (1) those that caused none or only mild disability (not clinically significant) and (2) those that caused moderate, very much, or extreme disability (clinically significant) (Mantri et al., 2020). DSM-5 defines a “disorder” as an impairment in social or occupational functioning that requires medical treatment (Roehr, 2013).

Receiver operator curve (ROC) analysis was performed to determine the best cut-off point for the MISS-HP. The cut-off point for the MISS-HP was determined based on the total score that was most sensitive and specific for identifying clinically significant functional disability. An accuracy of >50% was considered as acceptable (Chehrei et al., 2016).

##### Convergent validity

For convergent validity, the Pearson correlation coefficient was used to examine the correlation between the scores of the Persian version of MISS-HP, GHQ-12, and IES-R. As o higher scores of MISS-PH, GHQ-12, and IES-R show higher moral injury, mental disorders, and PTSD, respectively, convergent validity is confirmed in case of a positive correlation between MISS-HP, GHQ, and IES-R scores.

#### Reliability

Internal consistency was tested on 50 samples of healthcare professionals before construct validity (pilot study) and on 455 samples after factor analysis. To interpret the obtained coefficients, values equal or greater than 0.7 were considered to be acceptable reliability (Chehrei et al., 2016).

## Results

### Face validity

According to the participants, using health-care workers in the items was more understandable than health care professionals. In addition, two participants believed that the word “betrayed” in the first item was unclear. In this phase, we only replaced the health care professionals with health care workers. The Item Impact score of the items 8 and 11 was not acceptable; however, we did not delete any item for the next phase (Table 1).

**TABLE 1:** Face and content validities and internal consistency of the Persian Version of the Moral Injury Symptoms Scale-Health Care Professionals Version.

Item	Face validity (item impact) (*n* = 10)	Content validity index (*n* = 10)	Corrected item-total correlation (*n* = 50)	Cronbach’s alpha if item deleted (*n* = 50)
1. I feel betrayed by other health professionals whom I once trusted.	2.1	1	0.43	0.78
2. I feel guilty failing to save someone from being seriously injured or dying.	3.28	1	0.65	0.76
3. I feel ashamed about what I have done when providing care to my patients.	3.36	1	0.57	0.77
4. I feel ashamed about what I have not done when providing care to my patients.	3.36	1	0.60	0.76
5. I am troubled [upset, disturbed] by having acted in ways that violated my own morals or values.	3.36	1	–0.28	0.84
6. Most people with whom I work as a health professional are trustworthy.	2.8	1	0.58	0.76
7. I have a good sense of what makes my life meaningful as a health professional.	4.05	1	0.59	0.76
8. I have forgiven myself for what’s happened to me or to others whom I have cared for.	3.96	1	0.58	0.76
9. All in all, I am inclined to feel that I’m a failure in my work as a health professional.	1.16	0.9	0.60	0.77
10. I sometimes feel God is punishing me for what I have done while caring for patients.	2.73	1	0.68	0.76
11. I sometimes feel God is punishing me for what I have not done while caring for patients.	2.73	1	0.64	0.76
12. Compared to before I went through these experiences, my religious/spiritual faith has strengthened.	3.87	1	–0.08	0.82
13. Do the feelings you indicated above cause you significant distress or impair your ability to function in relationships, at work, or other areas of life important to you? In other words, if you indicated any problems above, how difficult have these problems made it for you to do your work, take care of things at home, or get along with other people?	1.24	1	0.44	0.79

### Content validity

In the qualitative evaluation of the content validity, the experts separated the items 3 and 9 i.e., “I feel ashamed about what I have done when providing care to my patients,” “I feel ashamed about what I have not done when providing care to my patients,” “I sometimes feel God is punishing me for what I have done while caring for patients,” “I sometimes feel God is punishing me for what I have not done while caring for patients.” As a result, at the end of this phase, the Persian version of the MISS-HP was a 13-item scale. The I-CVI of all items was above 0.9, while the S-CVI was 0.99 (Table 1).

### Pilot study

Fifty participants fulfilled the scale for assessing internal consistency. The Cronbach’s alpha coefficient for the MISS-HP was 0.79. The MISS-HP item-total correlations ranged from –0.28 (Item 5) to 0.68 (Item 10). The item-total correlations for 11 items of the MISS-HP were ≥ 0.43 (Table 1).

### Construct validity

#### Structural validity

The majority of the participants were younger than 30 years and the majority were female married nurses with less than 5 years of work experience (Table 2).

**TABLE 2:** The participants’ characteristics.

Variable	For exploratory factor analysis (*n* = 255)		For confirmatory factor analysis (*n* = 200)	
		
	Frequency	Valid percent	Frequency	Valid percent
Age (year.)*				
23–30	111	44.0	95	48.2
31–40	102	40.5	65	33.0
>40	39	15.5	37	18.8
**Gender**				
Male	43	16.9	45	22.5
Female	212	83.1	155	77.5
Marital status*				
Single	65	25.7	55	27.6
Married	186	72.9	142	71.4
Other	2	0.8	2	1.0
**Educational level**				
Practical nursing	4	1.6	4	1.0
B.Sc.	209	82.0	163	81.5
M.Sc.	22	8.6	19	9.5
MD	5	2.0	2	1.0
PhD/specialty	15	5.8	12	6.0
Major*				
Operating room	16	6.3	9	4.5
Nursing	188	73.7	146	73.4
Anesthesiology	21	8.2	17	8.5
Medicine	19	7.5	14	7.0
Midwifery	7	2.7	7	3.5
Laboratory	–	–	2	1.0
Nursing assistant	4	1.6	4	2.0
Work experience (year)*				
1–5	108	42.5	86	43.2
5.1–10	43	16.9	31	15.6
>10	103	40.6	82	41.2
**Hospital**				
A	89	34.9	63	31.5
B	78	26.7	62	27.5
C	68	30.6	55	31.0
D	20	7.8	20	10.0
Ward*				
Critical/intensive	61	24.7	44	23.0
Others	186	75.3	147	77.0
Monthly income (million Toman)*				
2–4	4	1.5	2	1.0
4–6	22	8.7	14	7.1
6–8	84	33.1	65	33.0
8–10	117	46.1	97	49.2
>10	27	10.6	19	9.7
History of mental disorders*				
Yes	19	7.5	15	7.5
No	234	92.5	185	92.5

*Missing value.

The percentage of missing responses and frequency of response options are present in Table 3. In general, the items were well-accepted, with percentages of missing per item ranging from 0.0 to 0.04%. There was no ceiling or floor effect for any item. The missing values were replaced with medians for conducting EFA.

**TABLE 3:** Data description and exploratory factor analysis of the Persian Version of the Moral Injury Symptoms Scale-Health Care Professionals Version.

Item	Missing value	Participants response to the MISS-HP (*n* = 255) (n/%)		Factor loading		
			
		Strongly disagree	Strongly agree	1	2	3
1. I feel betrayed by other health professionals whom I once trusted.	0	75 (29.4)	8 (3.1)	–	–	–
2. I feel guilt over failing to save someone from being seriously injured or dying.	0	95 (37.3)	13 (5.1)			0.58
3. I feel ashamed about what I have done when providing care to my patients.	0	162 (63.5)	5 (2.0)			0.51
4. I feel ashamed about what I have not done when providing care to my patients.	4	108 (43.0)	15 (6.0)			0.82
5. I am troubled [upset, disturbed] by having acted in ways that violated my own morals or values.	1	20 (7.9)	71 (28.0)			0.61
6. Most people with whom I work as a health professional are trustworthy.	2	6 (2.4)	37 (14.6)		0.75	
7. I have a good sense of what makes my life meaningful as a health professional.	1	8 (3.1)	71 (28.0)		0.75	
8. I have forgiven myself for what’s happened to me or to others whom I have cared for.	3	16 (6.3)	39 (15.5)		0.80	
9. All in all, I am inclined to feel that I’m a failure in my work as a health professional.	1	137 (53.9)	4 (1.6)	0.63		
10. I sometimes feel God is punishing me for what I have done while caring for patients.	2	155 (61.3)	1 (0.4)	0.90		
11. I sometimes feel God is punishing me for what I have not done while caring for patients.	2	143 (56.5)	3 (1.2)	0.87		
12. Compared to before I went through these experiences, my religious/spiritual faith has strengthened.	5	31 (12.4)	12 (4.8)	–	–	–
Eigen values				3.02	1.64	1.09
Explained variance				30.20	16.36	10.93
		**Not at all**	**Extremely**			
13. Do the feelings you indicated above cause you significant distress or impair your ability to function in relationships, at work, or other areas of life important to you? In other words, if you indicated any problems above, how difficult have these problems made it for you to do your work, take care of things at home, or get along with other people?	9	87 (35.4)	1 (0.4)			

Bartlett’s test of sphericity was used to determine if the sample size was appropriate for factor analysis and whether the data came from a normally distributed population. This test was statistically significant (χ^2^ = 761.34, df = 66, *P* < 0.001). The Kaiser-Meyer-Olkin (KMO) coefficient in this study was 0.694, confirming the factorability of the correlation matrix of the MISS-HP. PCA with Promax rotation, and a four-factor solution with an Eigen value > 1 was obtained. The four-factor solution using the PCA extraction method explained 60.51% of the data variance. According to this EFA, the item #1 had a cross loading and the item #12 had a negative loading; therefore, in the next step, the PCA with Promax rotation was conducted excluding these two items. According to the Eigen value, the items loading, and scree plot, a three-factor solution was extracted (Bartlett’s test of sphericity χ^2^ = 637.14, df = 45, *P* < 0.001; and KMO = 0.695) which explained 57.49% of the data variance (Table 3).

**FIGURE 1:**
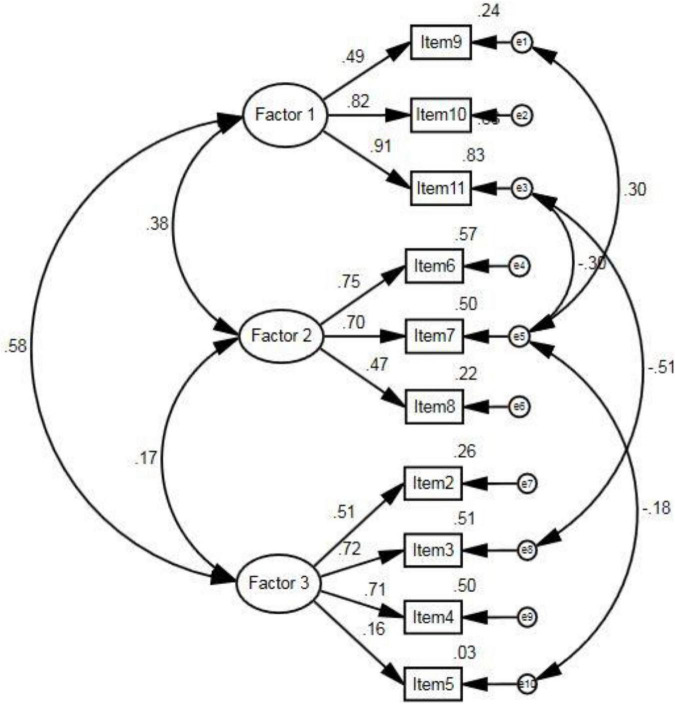
The confirmatory factor analysis of the Persian Version of the Moral Injury Symptoms Scale-Health Care Professionals Version.

Following the identification of a three-factor solution via EFA, CFA was used to further test the factor model that emerged from EFA. The models of first-order confirmatory factor analysis were used. Goodness-of-fit indices were used to determine the degree of fit between the data and the results of the hypothesized models. All of the factor loadings were significant except the item #5 (*t*-values > 1.96, *P* < 0.001). The χ^2^-associated *P* value was less than the significance level of 0.05 (χ^2^ = 48.945, d.f. = 28, and *P* = 0.008). All fit indices were acceptable (χ^2^/d.f. = 1.75, RMSEA = 0.06, GFI = 0.95, AGFI = 0.91, CFI = 0.96, IFI = 0.96, and NFI = 0.92) (Figure 1). Consequently, we could use the model to confirm the structure resulting from the exploratory factor analysis.

#### Cut-off point

**FIGURE 2:**
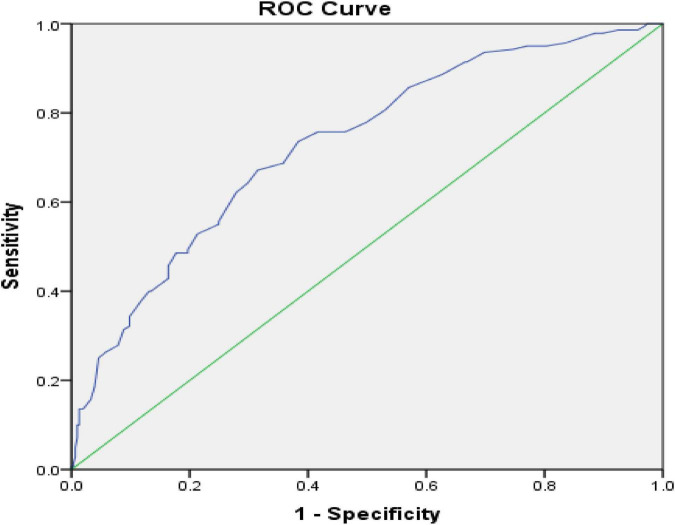
ROC Curve with Reference Line on the Persian Version of the Moral Injury Symptoms Scale-Health Care Professionals Version.

Of 455 participants, 10 samples were excluded due to the presence of missing value in item# 13, which was necessary to calculate the ROC curve, so ROC analysis was performed on 445 samples. Given that the Persian version of MISS-HP had 10 items, the resulting scores varied from 10 to 100. According to the original MISS-HP study, the answer to item# 13, (two subgroups without or with moderate-to-severe impact) was considered as the gold standard. Thus, the hypothesis was that individuals who scored higher on the MISS-HP had a positive result according to the gold standard. In other words, moral injury led to moderate to severe impact on their families and work performances. According to the ROC curve (Figure 2), the area below the curve, which is equal to the accuracy of the scale, was 0.73 (confidence interval 0.68–0.78, *P* < 0.001). Therefore, the accuracy of the MISS-HP scale is good.

The sensitivity and specificity of the scale were 67.1 and 68.5% at point 36.5, which was considered as the best positive point or cut-off point. Therefore, according to this cut-off point, a score ≥36.5 indicates moral injury, while a score lower than 36.5 indicates no moral injury.

#### Convergent validity

There was a positive and moderate correlation between the Persian version of MISS-HP, the GHQ (*r* = 0.34, *P* < 0.001), and IES-R scores (*r* = 0.40, *P* < 0.001).

### Reliability

The Cronbach’s alpha coefficient for the whole sample was (*n* = 455) 0.70. The MISS-HP item-total correlations ranged from 0.06 (Item# 5) to 0.54 (Item# 10). The item-total correlations for 9 items of the MISS-HP were ≥ 0.27.

## Discussion

The present study aimed to investigate the psychometric properties of the Moral Injury Symptoms Scale- Health Care Professionals (MISS-HP) in Iran. The Persian version of the MISS-HP contains 10 items that, unlike the original version, assesses guilt, shame, moral concerns, loss of trust, loss of meaning/purpose, difficulty forgiving, self-condemnation, and feeling punished by God. Item 11 is not a main item of the scale, but it is mentioned only to investigate the impact of moral injury on one’s performance. The responses are scored from strongly disagree (1) to strongly agree (10). Therefore, the range of scores varies from 10 to 100. The cut-off point of the Persian version is 36.5, meaning that people who obtain a score equal to or higher than 36.5 have symptoms of moral injury. It should be noted that the cut-off point of the original version is 36. The study results showed acceptable psychometric properties of the Persian version of MISS-HP. A review of the literature showed that due to the COVID-19 outbreak and the possibility of moral injury of health care professionals, the Chinese (Zhizhong et al., 2020), German (Trifunovic-Koenig et al., 2022), Turkish (Üstün, 2022), and the United States (Mantri et al., 2020) versions of this questionnaire have been used and measured psychometrically.

### Content validity

Validity refers to the accuracy of a method or tool in measuring a particular feature. In qualitative evaluation of content validity in this study, experts separated items 3 “I feel ashamed about what I have done or not done when providing care to my patients” and 9 “I sometimes feel God is punishing me for what I have done or not done while caring for patients” from each other. As the phrase “done or not done” are used separately in Persian, experts suggested dividing these items into two parts and items 3 and 9 each included 2 separate items. Therefore, the Persian version of MISS-HP became a 12-item scale at this stage. However, items 1 “I feel betrayed by other health professionals whom I once trusted” and 12 “compared to before I went through these experiences, my religious/spiritual faith has strengthened” were not confirmed in structural validity. Therefore, the final Persian version of MISS-HP became a 10-item scale. These two items were rejected because they contained the religious beliefs of healthcare professionals. In addition, item 10 in the German version was removed because it dealt with the beliefs in God and 25% of all healthcare providers in Germany are atheists/agnostics or not affiliated with religious groups (Trifunovic-Koenig et al., 2022). In order to measure and evaluate this questionnaire better, spiritual, religious and cultural differences of the health care professionals should be considered in future research. In the present study, the I-CVI and S-CVI of all items were 0.9 and 0.99, respectively. According to Polit and Beck, values equal to or higher than 0.90 are acceptable for S-CVI. Furthermore, the CVI scores of all items in the Turkish version of the MISS-HP were greater than 0.80 (Üstün, 2022). Content validity was not reported in the German, Chinese, and the United States versions.

### Construct validity: Structural validity

In the present study, there was no ceiling or floor effect for any item. Bartlett’s sphericity test was used to check the structural validity and it was found that the sample size was suitable for factor analysis and the data were obtained from a normally distributed population. The KMO coefficient in this study was 0.69, confirming the factorability of the correlation matrix of the Persian version of MISS-HP. According to Eigen value, item loading, and scree plot, a three-factor structure was extracted. The study results indicated that Iranian health care professionals obtained three acceptable factors for the MISS-HP that explained nearly 60% of the variance. The variance obtained in the present study was higher than that reported in the German (Trifunovic-Koenig et al., 2022), Chinese (Zhizhong et al., 2020), and the United States versions (Mantri et al., 2020). In the Chinese version of the MISS-HP, EFA suggested three factors that accounted for 59% of the variance (Zhizhong et al., 2020). In the United States version of the MISS-HP, three factors were identified, which explained 56.8% of the variance (Mantri et al., 2020). However, in the Turkish version of the MISS-HP, the mean variance was 84.48% (Üstün, 2022), which was higher than that in the present study.

After analysis of the MISS-HP in the present study, a three-factor structure with 10 acceptable items was obtained. In line with the results of the present study in the original version (in the United States version) of the MISS-HP, three factors with 10 items were identified (Mantri et al., 2020). A three-factor structure with 10 items was defined in the Turkish version of the MISS-HP (Üstün, 2022). However, the correlation between items and modified scales for an item was not acceptable in the German version of the MISS-HP. The German version was approved with 9 items and three factors (Trifunovic-Koenig et al., 2022). The Chinese version of the MISS-HP suggested three factors (Zhizhong et al., 2020). Therefore, there may be small differences due to translation or cultural differences in item interpretation.

### Cut-off point

The cut-off point of the MISS-HP in the present study was a score ≥36.5, which showed moral injury, with a sensitivity of 67.1% and a specificity of 68.5%. In line with the results of the present study in the original version (the United States) of the MISS-HP, a cut-off point of 36 or higher was considered for the diagnosis of MI symptoms, which sensitivity was 84% for diagnosis of symptoms causing functional disability (Mantri et al., 2020). However, the cut-off point of the Turkish version of the MISS-HP was 46, which had a prediction accuracy of 90% (Üstün, 2022). The cut-off point of the Chinese version of the MISS-HP was 50 with a correct prediction of 71% (Zhizhong et al., 2020). In addition, the cut-off point of the German version of the MISS-HP was 28.5 (sensitivity 89% and specificity 63%) (Trifunovic-Koenig et al., 2022). Cultural diversity and different healthcare systems may affect the concept of moral injury, indicating different cut-off points, which should be considered in future studies. However, the cut-off point determined in the present study, which is close to the main one, is acceptable for determining the MISS-HP in the Iranian community.

### Construct validity: Convergent validity

The current study showed a positive and moderate correlation between the scores of the Persian version of MISS-HP, the GHQ, and IES-R. Consistent with the results of the present study, Wang et al. (2022) showed a positive correlation between MISS-HP scores, depression, anxiety, low well-being, and burnout during the COVID-19 outbreak in China (Wang et al., 2022). In addition, Zerach and Levi-Belz (2022) showed a correlation between symptoms of moral injury (MI) and symptoms of complex post-traumatic stress disorder (CPTSD) during the COVID-19 outbreak in Israel (Zerach and Levi-Belz, 2022). The Turkish version of the MISS-HP indicated a significant positive correlation between the Compassion Fatigue Short-Scale (CF-SS) and the MISS-HP scores (Üstün, 2022). Convergent validity was shown with a 4-item scale (*r* = 0.45 for physicians, *r* = 0.43 for nurses) in the Chinese version of the MISS-HP. There was also a moderate correlation between MISS-HP, burnout, well-being (*r* = 0.34–0.47), and symptoms of depression and anxiety (*r* = 0.37–0.45) (Zhizhong et al., 2020). In the United States version, the MISS-HP had a poor to moderate correlation with the symptoms of religiosity, depression, and anxiety (*r* = 0.25–0.37), while it had a strong correlation with physicians’ burnout (*r* = 0.57) and a multi-item criterion of MI symptoms (*r* = 0.65) (Mantri et al., 2020). The convergent validity of the German version of the MISS-HP showed a positive correlation between the G-MISS-HP and G-SVESTR subscales (Trifunovic-Koenig et al., 2022).

### Internal consistency

In the present study, Cronbach’s alpha of 0.70 was obtained for the Persian version of the MISS-HP. The total-item correlation of the MISS-HP ranged from 0.06 to 0.54. Consistent with the study results, the Cronbach’s alpha of the Chinese version of the MISS-HP was acceptable for both nurses and physicians (nurses = 0.71 and physicians = 0.70). In addition, test-retest reliability was from 0.41 for items to 0.74 for the whole scale (Zhizhong et al., 2020). However, the Cronbach’s alpha reported in the Turkish (Cronbach’s alpha = 0.91) (Üstün, 2022), the German (Cronbach’s alpha = 0.79) (Trifunovic-Koenig et al., 2022) and the United States versions (Cronbach’s alpha = 0.75) (Mantri et al., 2020) were higher than that in the present study. It is necessary to pay attention to the type of healthcare groups studied when interpreting the results.

Demographic characteristics change rapidly, and health care systems will face global crises and challenges, such as the COVID-19, which can lead to many problems such as anxiety, stress, anger, and psychological problems among health care workers. These social and occupational changes are leading healthcare workers to new moral challenges. As a result, healthcare professionals may experience more ethical conflicts and errors, which put employees at risk for moral injury. Managers and psychotherapists require a comprehensive tool to assess this problem. The MISS-HP can be very helpful in addressing the challenges of healthcare systems.

## Study limitations

This study provided a useful tool for measuring moral injury among health care professionals in Iran. By reviewing this questionnaire in larger communities, we can gain a better understanding of the effectiveness of this questionnaire in clinical practice. Although the present study used a standard translation method to develop an Iranian version of the MISS-HP, cultural differences between Iranian and Western societies (where the scale was originally developed and designed) might influence the results. Furthermore, self-report nature of the questionnaire is another factor that should be considered. Different geographical regions and cultures in Iran are other important factors that should be considered when reporting the results. Using larger samples of different health care professionals in different Iranian contexts and geographies can help strengthen our results.

## Conclusion

This study found that the MISS-HP is a concise, comprehensive, valid and reliable questionnaire for assessing moral injury among health care professionals in Iran. The solid psychometric properties of the Persian version of MISS-HP confirm its usefulness in measuring moral injury among health care professionals. However, this questionnaire needs to be tested on larger groups and people from different cultures. This will help managers and researchers improve the mental health of health care professionals.

## Data availability statement

The raw data supporting the conclusions of this article will be made available by the authors, without undue reservation.

## Ethics statement

The studies involving human participants were reviewed and approved by the Kerman University of Medical Sciences. The patients/participants provided their written informed consent to participate in this study.

## Author contributions

AM and MZ designed the study and collected data. HK and MD contributed to the study design, they provided critical feedback on the study and analysis, and inputted to the draft of this manuscript. AM and MZ wrote the manuscript. All authors have read and approved the final manuscript.
